# Synbiotic supplementation leads to gut microbiome communities with functional capacity linked to better brain white matter development in preterm infants

**DOI:** 10.1080/19490976.2026.2743949

**Published:** 2026-10-07

**Authors:** Anna Voulgari-Kokota, Els Janson, Ineke Heikamp de Jong, Jan Knol, Ruurd van Elburg, Niek E. van der Aa, Lisa M. Hortensius, Jeroen Dudink, Caroline G. M. de Theije, Lidewij Schipper, Marta Kozior, Monique van de Lagemaat, Floris Groenendaal, Frank van Bel, Annemieke Wildt-Grootendorst, Sandra Drost-Verhoef, Annelies Hennink, Charlie C. Obihara, Jacqueline L. A. M. van Hillegersberg-Schilder, Carin M. Dassel, Johanna H. Oudshoorn, Clemens B. Meijssen, Inge P. de Boer, Károly E. Illy, Nathalie Claessens, Max A. Viergever, Ivana Isgum, Sudarshan Shetty, Harm Wopereis, Manon Benders, Clara Belzer

**Affiliations:** a Laboratory of Microbiology, Wageningen University & Research, Wageningen, The Netherlands; b Department of Neonatology, Wilhelmina Children's Hospital, University Medical Center Utrecht, Utrecht University, Utrecht, The Netherlands; c University Medical Center Utrecht Brain Center, Utrecht University, Utrecht, The Netherlands; d Danone Research & Innovation, Utrecht, The Netherlands; e Department of Pediatrics, Emma Children's Hospital, Amsterdam University Medical Center, Amsterdam, The Netherlands; f Department for Developmental Origins of Disease, University Medical Center Utrecht Brain Center and Wilhelmina Children's Hospital, Utrecht University, Utrecht, The Netherlands; g Department of Pediatrics, Elisabeth-Tweesteden Hospital, Tilburg, The Netherlands; h Department of Pediatrics, Sint Antonius Hospital, Nieuwegein, The Netherlands; i Department of Pediatrics, Deventer Hospital, Deventer, The Netherlands; j Department of Pediatrics, Gelre Hospital, Apeldoorn, The Netherlands; k Department of Pediatrics, Meander Medical Centre, Amersfoort, The Netherlands; l Department of Pediatrics, Diakonessenhuis, Utrecht, The Netherlands; m Department of Pediatrics, Hospital Rivierenland, Tiel, The Netherlands; n Image Sciences Institute, University Medical Center Utrecht, Utrecht, The Netherlands; o Department of Biomedical Engineering & Physics, Amsterdam University Medical Center, Amsterdam, The Netherlands; p Informatics Institute, University of Amsterdam, Amsterdam, The Netherlands; q Department of Radiology & Nuclear Medicine, Amsterdam University Medical Center, Amsterdam, The Netherlands

**Keywords:** Probiotics, prematurity, preterm infants, preterm microbiome, brain maturation, gut–brain, *Bifidobacterium breve*

## Abstract

Preterm birth, a major cause of brain injury, is often linked to dysregulated gut microbiome development. This association underscores microbial metabolic function as a modifiable target to support neurodevelopment. In this secondary analysis of data derived from a randomized controlled trial (Trial Registration: ISRCTN96620855), we tested whether daily nutritional supplementation with *Bifidobacterium breve* M-16V, short- and long-chain oligosaccharides, and L-glutamine could steer the gut microbiome of very and extremely preterm infants toward communities that support brain maturation. The gut microbiome was profiled with longitudinal shotgun metagenomics at nine time points during the intervention, which started at 48–72 h after birth and continued until 36 weeks postmenstrual age. Additionally, MRI scans were conducted when infants reached term-equivalent age to evaluate brain maturation. Supplementation promoted the early establishment of *Bifidobacterium*-rich communities in the test group, with enhanced capacity for amino acid biosynthesis and pyruvate fermentation towards acetate and lactate production. Integration of microbiome data with brain developmental markers *post hoc* showed that the same functions were markedly reduced in infants with delayed white-matter myelination. By integrating microbiome functional capacity profiling and evaluation of brain maturation via MRI, this study demonstrated that early microbial modulation could influence brain development, positioning the preterm gut microbiome as a clinically actionable target.

## Introduction

It is estimated that 11% of all births globally are preterm, with two-thirds occurring without an evident risk factor.^1^ Despite advances in perinatal and postnatal care, preterm birth remains strongly associated with long-term neurodevelopmental disorders that can translate into behavioral and cognitive difficulties later in life,^2^ ^,^ ^3^ as it disrupts a critical *in utero* developmental period marked by fundamental brain maturation processes.^4-7^

For preterm infants, the fundamental brain maturation processes occurring in early life coincide with the assembly of their gut microbiome. Emerging gut microbiome imbalances may disrupt the developmental trajectories of their highly sensitive neural circuits^8-10^ and even trigger the onset of severe conditions linked to neurodevelopmental disorders, such as neonatal sepsis and necrotizing enterocolitis.^11-14^ Conversely, the timely establishment of a beneficial gut microbiome may support brain maturation through improved metabolic output and gut–brain signaling pathways.^15^

Because preterm infants miss key periods of in-utero growth, targeted early nutrition becomes especially important – it not only supports brain development but also helps them “catch up” to the physiological and microbial trajectories typically seen in healthy term infants. In full-term, vaginally-delivered infants, early gut colonization typically originates from maternal microbial communities enriched with beneficial *Bifidobacterium* spp.^16^ In preterm infants, however, the initial gut microbiome communities are primarily associated with gestational age, regardless of delivery mode.^17-21^

More specifically, during the first days of life, aerobes such as Staphylococci predominate in the immature preterm infant gut, with *Enterobacteriaceae* becoming more abundant at later postmenstrual ages.^19^ ^,^ ^20^ With gut maturation, decreasing proportions of Bacilli may be accompanied by increasing proportions of strict anaerobes such as Clostridia.^21^ The establishment of a *Bifidobacterium*-dominated community, which is characteristic of healthy neurodevelopment, is significantly delayed, possibly due to high pre-existing levels of *Enterobacteriaceae*.^21-25^

In the postnatal period, breastfed, full-term infants typically receive a continuous supply of maternal microbes and human milk-derived prebiotics that promote the early enhancement of *Bifidobacterium* spp.^26-28^ In contrast, preterm infants often exhibit impaired feed digestion due to immature gastrointestinal function, reduced enzyme activity, and delayed motility.^29^ ^,^ ^30^ These limitations can hinder their ability to fully utilize human milk components – especially complex oligosaccharides that normally fuel *Bifidobacterium* growth – thereby slowing the establishment of beneficial microbial communities. Moreover, preterm infants are frequently exposed to clinical practices associated with neonatal intensive care units (NICUs), such as early exposure to antibiotics, which can further delay microbiome maturation, often because of prolonged carriage of multidrug-resistant *Enterobacteriaceae*.^31^ ^,^ ^32^

Optimizing nutrition that stimulates early gut microbial colonization in preterm infants is a promising therapeutic strategy for accelerating microbiome maturation and ultimately lowering neurodevelopmental risk by influencing gut–brain signaling and energy availability during critical windows of brain development.^33^ Recently, it has been suggested that enhanced microbial metabolic capacity may support neuroprotection and improved developmental outcomes in preterm infants.^34^ Also, observational and randomized studies have investigated the potential of probiotics to prevent inflammatory diseases that affect neurodevelopment, such as sepsis and necrotizing enterocolitis, with findings ranging from no observed benefit^35^ to general risk reduction.^36^

Differences in the reported effects of probiotics on preterm infant health likely reflect differences in the probiotic strains used, the simultaneous use or absence of prebiotics, varying sample sizes, and clinical practices or study designs.^37^ Furthermore, no randomized trial to date has directly evaluated the effect of controlled enteral supplementation with pro- and prebiotics on gut colonization, microbiome function, and subsequent brain development in preterm infants.

The present study uses longitudinal metagenomic data from a cohort of extremely and very preterm infants derived from a randomized controlled trial (Trial Registration: ISRCTN96620855),^38^ that tested the impact of daily enteral supplementation with probiotics, prebiotics, and L-glutamine on brain development assessed at term-equivalent age (TEA). The strain *Bifidobacterium breve* M-16V was selected for its established safety and prior use in NICUs for preterm and low-birth-weight infants, where it had demonstrated potential benefits for the gut microbiome development.^39^ ^,^ ^40^ L-glutamine was included for its role in reducing neonatal infections and gastrointestinal dysfunction,^41^ ^,^ ^42^ and a prebiotic mix of short-chain galactooligosaccharides and long-chain fructooligosaccharides (scGOS/lcFOS) for their prebiotic properties.^43^ We hypothesized that the nutritional intervention would support the establishment of *Bifidobacterium*-rich gut communities and would accelerate functional maturation of the gut microbiome in preterm infants. Furthermore, we investigated whether shifts in the microbial metabolic capacity were linked to improved brain developmental markers assessed at TEA. The randomized intervention, implemented within a clearly defined time window, provided a unique opportunity to examine how targeted early nutritional modulation influences gut microbiome assembly during a critical developmental period. Randomization helps minimize potential confounding, while repeated longitudinal sampling enables detailed characterization of microbial trajectories and their potential relationship with brain development at term-equivalent age.

## Materials and methods

### Objectives

A double-blind, randomized, controlled, parallel-group and single-center intervention study was conducted to test the combined effect of the probiotic strain *B. breve* M-16V, a prebiotic mix of scGOS/lcFOS, and the amino acid L-glutamine on the postnatal gut microbiome and brain development of infants born between 24 and 30 weeks of gestation.^38^ The present study explores secondary objectives of the trial as described in the study protocol,^38^ with the main outcome presented here being the composition and temporal development of the gut microbiome in preterm infants assessed with shotgun metagenomics sequencing. Post hoc analyses examined associations of microbiome structure and function with brain injury markers assessed with MRI.

## Ethical approval, study design, and participants

Ethical approval was obtained by the Medical Ethical Committee of the University Medical Center Utrecht (UMCU) (reference: 5-213/M NL 49902.041.14). The study was performed in accordance with the principles stated in the Declaration of Helsinki. Details on trial registration are available online: https://www.isrctn.com/ISRCTN96620855.

The trial began at the level III NICU of the UMCU and continued in seven Dutch regional hospitals for infants transferred before completing 36 weeks postmenstrual age (PMA). Custodial parents of 338 eligible infants were approached shortly after admission to the obstetric ward or NICU with signs of preterm labor (<30 weeks of gestation). Following written free and informed consent, infants who satisfied the inclusion criteria were randomized 1:1 to either the intervention or control group of the study with permuted block randomization stratified for sex (male and female), using the PLAN procedure of *SAS* v4.3. From October 2018 to May 2022, 99 infants were randomized to the intervention or control group of the trial. The assessors, parents, and clinical staff remained blinded at least until the last participant reached 24 months of corrected age. The flow of participants through enrollment, randomization, intervention allocation, follow-up, and inclusion in the analyses is demonstrated in Figure S1, and their demographic and clinical characteristics are shown in Table 1. The number of available samples per time point is summarized in Table S1, and the demographic and clinical characteristics of participants sampled at each time point are summarized in Table S2.

**Table 1.** Demographic and clinical characteristics of participants.

Parental consent and inclusion criteria fulfilled: 99		
Assignment to groups	Intervention: 50	Control: 49
Infants with metagenomes available: 84		
Assignment to groups	Intervention: 44	Control: 40
Sex: male (%)	63.63, CI: 49.9–75.7	60.00, CI: 57.2–84.8
Race: white (%)	90.90, CI: 78.3–97.5	84.62, CI: 69.5–94.2
Vaginal delivery (%)	45.45, CI: 30.4–60.4	42.50, CI: 27.2–58.8
Nutrition with > 80% mother milk (%)	72.72, CI: 57.2–84.8	72.50, CI: 56.1–85.4
Weeks of gestation (average)	27.76, CI: 27.34–28.18	27.46, CI: 26.99–27.93
Birth weight in grams (average)	1005.18, CI: 929–1081	1070.00, CI: 981–1159
Infants with both metagenomes and Kidokoro scores available: 76		
Assignment to groups	Intervention: 39	Control: 37
Sex: male (%)	64.10, CI: 48.8–77.8	62.16, CI: 45.5–77.1
Race: white (%)	89.74, CI: 75.8–97.1	86.11, CI: 70.5–95.3
Vaginal delivery (%)	46.15, CI: 30.4–62.6	37.83, CI: 22.5–54.9
Nutrition with > 80% mother milk (%)	71.79, CI: 55.1–84.9	70.27, CI: 53.0–84.1
Weeks of gestation (average)	27.72, CI: 27.27–28.17	26.97–27.95
Birth weight in grams (average)	1007.48, CI: 924–1091	1068.78, CI: 972–1166

The exclusion criteria included any proven or suspected metabolic disorder, genetic syndrome, congenital central nervous system malformation or infection, gastrointestinal malformation, use of gastric acid inhibitors, poor survival prognosis (at supervising clinician’s discretion), admission to another hospital, and participation in other postnatal intervention studies. Participation was discontinued in cases of health risk (informed at the supervising clinician’s discretion), consent withdrawal by the custodial parents, and suspected or proven inability to comply with the study protocol.

The intervention began within 48–72 h after birth and continued until 36 weeks PMA or discharge home, whichever came first. The intervention group received a daily enteral dose of *B. breve* M-16V via nasogastric tube, orogastric tube, or syringe. The starting daily dose was 3 × 10^9^ or 1.5 × 10^9^ colony-forming units (CFU), for birth weight higher or lower than 1000 g, respectively. This dosage was increased to 3 × 10^9^ CFU when the daily enteral feeding intake reached 50  ml/kg. The control group received a daily dose of 0.6 or 0.3 g maltodextrin as a placebo, to match the weight of the test product. Additionally, the intervention group received a daily supplement to the regular milk feed with a starting dose of 0.05 g/kg L-glutamine and 0.1 g/kg scGOS/lcFOS (9:1), which increased to 0.3 g/kg and 0.6 g/kg, respectively, at full enteral feeding. The control group received an isocaloric placebo consisting of maltodextrin, casein, and whey protein hydrolysates at corresponding doses (0.5 g/kg/day initially and 3.0 g/kg/day at full enteral feeding). The placebo formulation was designed to match the carbon and nitrogen contents of the intervention supplement.

### Microbiome profiling

The first stool samples from infants were collected within 72 h after birth (meconium) and 1, 3, and 6 weeks after birth. Up to five additional samples were collected until hospital discharge or TEA, and up to three more samples were collected at follow-up visits (Table S1). The samples were stored at −70 °C, and whole genomic DNA was extracted with a Maxwell® RSC, Wisconsin, U.S.A., Genomic DNA Kit according to the manufacturer's cell pellet protocol, following sample homogenization, including pellet washing, removal of organic material and lipids, proteinase K digestion, and chemical lysis, at the Laboratory of Microbiology at Wageningen University. The extracted DNA was stored immediately at −20°C. Quality control, library preparation, and metagenomic shotgun sequencing were performed by GenomeScan B.V., Leiden. After quality control, the samples were retained if DNA ≥ 25  μl, OD260/280 ~1.8–2.0, and OD260/230 ~2.0–2.2. Samples of sufficient quality were then included in library preparation and sequenced with an Illumina NovaSeq 6000 (PE150). After quality filtering of the stool DNA, 473 metagenomes from 84 infants were obtained and included in all downstream microbiome analysis.

### Brain MRI scans

The infants were scanned at TEA on a 3.0 Tesla MR system (Achieva, Philips Medical Systems, Best, the Netherlands) with a 32-channel SENSE head coil. Diffusion, T1-, and T2-weighted imaging were performed as previously described.^44^ Mean fractional anisotropy (FA) was measured in a region of interest in the right posterior limb of the internal capsule (rPLIC) and in a tract segment of the right corticospinal tract (rCST), using whole-brain tractography (FA threshold > 0.1, curvature < 45°). Brain injury and maturation were assessed using Kidokoro scores by UMCU neonatologists.^45^ The Kidokoro system includes brain injury and maturation markers for white matter (cystic lesions, focal signal abnormalities, delayed myelination, ventricular dilation, and corpus callosum thinning), gray matter (gyral maturation delay, enlargement of extra-axial spaces, signal abnormalities), and cerebellum (volume reduction and signal abnormalities). Data was entered in an electronic Case Report Form by trained staff in line with data privacy laws. Of the 84 infants with longitudinal microbiome data available, 76 had usable brain MRI scans and evaluated Kidokoro scores for brain development and injury.

### Data analysis

Metagenomic sequence reads derived from stool sample DNA were quality filtered and trimmed with *Trimmomatic* v0.39 (parameters: ILLUMINACLIP:2:30:10, LEADING:3, TRAILING:3, SLIDINGWINDOW:4:15, MINLEN:36).^46^ Host DNA was removed with *Bowtie2* v2.5.4^47^using the publicly available Genome Reference Consortium h38 human genome assembly. *B. breve* M-16V strain was identified using the available genome (NCBI BioProject: PRJNA482528) and *StrainPhlAn* v4.1.0^48^ with clade-specific marker genes generated with *MetaPhlAn* v4.1.0.^49^ Taxonomic and functional profiling of the metagenomes were conducted with *MetaPhlAn* v4.1.0^49^ and *HUMAnN3* v3.9, respectively.^50^

Relative abundances of microbial taxa were compared between the control and the intervention group within each sampling point with Wilcoxon rank sum tests with Benjamini–Hochberg adjustment for false discovery rate control, and significance cutoff *P* < 0.05 in the native R *stats* package.^51^ Microbiome beta diversity within each sampling point was visualized using non-metric multidimensional scaling (NMDS) and tested with non-parametrical ANOSIM^52^ to assess the influence of the intervention (intervention/control), sex (male/female), enteral nutrition type (>80% mother milk, >80% preterm formula or combination), delivery mode (vaginal delivery/caesarian section), antibiotics (administration at time or within 1 week prior to sampling/no administration) and gestational age (24–26, 26–28, and 28–30 weeks).

To identify taxonomic balances in each sampling point predictive of the intervention or the control group and also of brain developmental markers (Kidokoro scores^45^), we used *selbal*, selecting models with probability AUC-ROC > 0.7.^53^ We used *indicspecies* to measure the specificity of microbial taxa (*indicVal*) to the same sample groups, using 10,000 permutations to assess confidence and significance cutoff *P* < 0.05.^54^

For exploring differences in microbial metabolic function and in specific metabolic pathways between sample groups, we performed differential abundance analysis using *DESeq2* with exclusion of pathways with average abundance lower than 1000 counts per million and prevalence < 10% across all samples, default internal normalization, dispersion estimation, Benjamini-Hochberg adjustment and significance cutoff *P* < 0.05.^55^ To test if FA measurements were associated to relative abundances of microbial taxa, we used Spearman rank correlations and significance cutoff *P* < 0.05 in *stats*.^51^ Microbiome clustering into community types was done with Ward hierarchical unsupervised method^56^ and checked using *pvclust* with 10,000 bootstrap replicates and acceptance threshold *P* < 0.05.^57^ The independence of the community types to the intervention and to brain developmental markers was tested with chi-squared tests and independence residual-based models using *vcd* and default settings.^58^

## Results

### Microbiome differences between the control and intervention groups

The relative abundance of *B. breve* M-16V was higher in the intervention group only at week 1, 3, and 6 after birth (Wilcoxon *P* = 1.76 × 10^−10^, for week 1, and *P* = 0.0002 for week 3 and 6) (Figure S2). The strain was detected (relative abundance > 0.5%) in 97.3%, 100%, and 93.94% of the infants of the intervention group, compared to 9.1%, 44.44%, and 39.39% of the control group infants at the same time points (Supplement 2). Total *B. breve* relative abundance, regardless of strain, was higher in the intervention group only at week 1, 3, and 6 after birth (Wilcoxon *P* = 1.1 × 10^−8^, *P* = 0.01, *P* = 0.033) (Figure S2). *B. breve* detection not attributed to the administered strain was detected in 9,1%, 11,11%, and 24,24% of the control group infants at the same time points, in comparison to 6,1% of the intervention infants only at week 6.

The overall microbial community (Figure S3) differed between groups at week 1, 3, and 6 (R = 0.4, *P* < 0.001; R = 0.10, *P* < 0.001, and R = 0.06, *P* = 0.003, respectively) (Figure 1A). No significant differences in the gut microbiome community structure were attributed to sex (male or female), nutrition (fed with > 80% mother milk, > 80% preterm formula or mix of the two), delivery mode (vaginal delivery or cesarian section) or gestational age (24–16, 26–28, or 28–30 weeks) at any time point; neither for all samples combined nor for samples of each intervention group separately. Antibiotic administration had a significant effect on the microbiome composition only at week 3 (R = 0.23, *P* = 0.030), with 9 out of 73 infants with available samples receiving antibiotics. At that time point, the relative abundance of *Enterobacter* was higher in subjects receiving antibiotics (Wilcoxon *P* = 0.002). The difference was significant for the control (Wilcoxon *P* = 0.008) but not for the intervention group (Wilcoxon *P* = 0.055) (Figure S4). Finally, no infant weight development difference was linked to the intervention (Figure S5).

**Figure 1.**
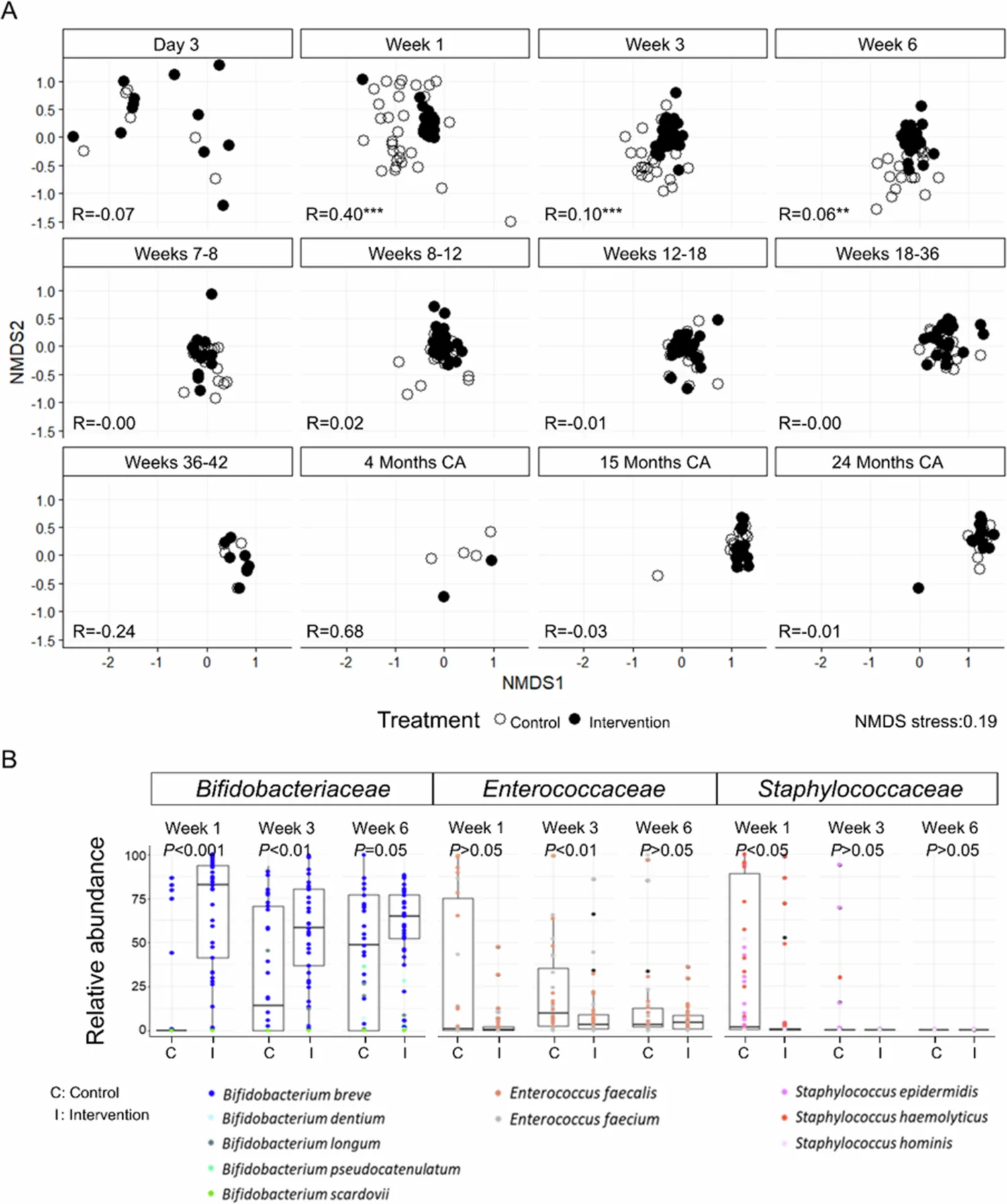
(A) NMDS ordination for preterm gut microbiome composition at the species level, faceted for every sampling time point. White represents the control group and black the intervention group. Community differences between groups were tested with ANOSIM: R stands for the ratio of variance explained, and asterisks indicate statistical significance (*P* < 0.001***, *P* < 0.01**). (B) Relative abundance of the bacterial families showing significantly different levels between the control (C) and the intervention (I) group, at week 1, 3, and 6 after birth. Boxplots represent the overall family abundance with the horizontal line at the median value. The colored circles represent the relative abundance of individual bacterial species within the family. *P-*values result from Wilcoxon rank sum tests, comparing family abundances between groups.

Comparison between the control and the intervention group showed higher *Bifidobacterium* spp. relative abundance from weeks 1 through 6 in the intervention group (Wilcoxon *P* = 7.9 × 10^−10^, *P* = 0.006, and *P* = 0.042, for week 1, 3, and 6, respectively), accompanied by lower *Staphylococcus* spp. (*P* = 0.017) at week 1 and lower *Enterococcus* spp. abundance (*P* = 0.008) at week 3 (Figure 1B). For the same period, microbiome clustering grouped all the microbial metagenomes into six community types (CTs) (Figure S6): CT-1: *Bifidobacterium*, CT-2: *Bifidobacterium-Klebsiella*, CT-3: *Enterobacter-Enterococcus-Escherichia*, CT-4: *Klebsiella*, CT-5: *Enterococcus*, and CT-6: *Staphylococcus*.

### Microbiome differences associated with brain development

No significant differences in the assessed brain injury markers were observed between the intervention and control groups (chi-squared analysis, *P* > 0.05). Two white matter injury markers were associated with differences in the gut microbial composition. The gut communities at week 1 differed between infants with (*n* = 11) and without (*n* = 52) white matter thinning of the corpus callosum at TEA (R = 0.20, *P* = 0.017), and the gut communities at week 3 differed between infants with (*n* = 20) and without (*n* = 45) white matter myelination delay at TEA (R = 0.25, *P* = 0.001). The other Kidokoro scores and FA values were not significantly associated with microbiome differences.

Community type transitions from the 1st to the 3rd week after birth were not independent of the occurrence of white matter thinning of the corpus callosum and white matter myelination delay at TEA (X^2^ = 1300.9, df = 48, *P* = 2.2 × 10^−18^). Transition to CT-3: *Enterobacter-Enterococcus-Escherichia* associated with white matter myelination delay and grouping under CT-6:*Staphylococcus* to thinning of the corpus callosum. Also, community type transitions linked to the intervention group were negatively associated with both types of white matter brain injury (Figure S6).

### Association of bacterial species to the synbiotic supplementation and to brain development markers

The balance of *B. breve* to *Staphylococcus epidermidis* and *Escherichia coli* for week 1 (AUC-ROC = 0.95) and to *S. epidermidis* and *Klebsiella michiganensis* at week 3 (AUC-ROC = 0.79) distinguished the intervention group from the control group (Figure 2), with higher levels of *B. breve* characterizing the intervention group. Additionally, a higher *Staphylococcus haemolyticus* to *B. breve* ratio at week 1 was indicative of white matter thinning of the corpus callosum (AUC-ROC = 0.75), and a higher *Enterobacter roggenkampii* to *B. breve* ratio at week 3 was indicative of white matter myelination delay (AUC–ROC = 0.78) (Figure 2).

**Figure 2.**
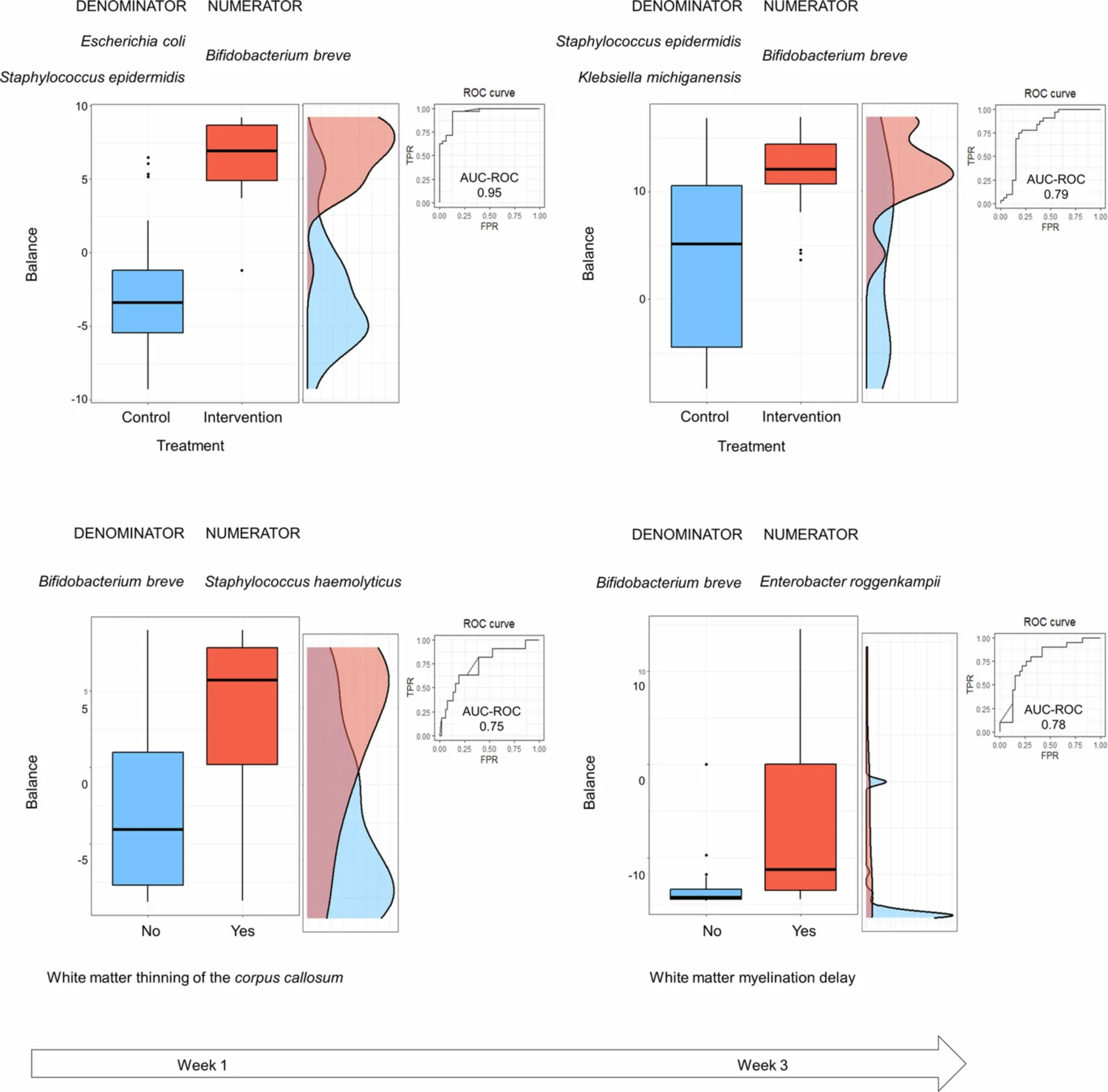
Gut microbiome compositional balance at week 1 and 3 after birth indicative of the intervention and the occurrence of white matter thinning of the corpus callosum or myelination delay. The box plots represent the distribution of the balance scores for the subject subgroups. The right part of each figure contains the ROC curve with an AUC-ROC value assessing the model’s performance and probability.

Indicator species analysis confirmed the specificity of *B. breve* to the intervention group for week 1 (IndicVal = 0.67, *P* = 0.001) and week 3 (IndicVal = 0.30, *P* = 0.015). It also confirmed the specificity of *B. breve* for infants not showing white matter injury at TEA for week 1 (IndicVal = 0.37, *P* = 0.035), as well as for week 3 (IndicVal = 0.42, *P* = 0.002). Furthermore, it confirmed the association of white matter thinning of the corpus callosum with *S. haemolyticus* (IndicVal = 0.50, *P* = 0.001) and of myelination delay with *E. roggenkampii* (IndicVal = 0.24, *P* = 0.007) (Table S3) for the same sampling points.

### Differences in the microbial functional capacity as a result of the intervention

From the 1st to the 3rd week after birth, several metabolic pathways related to amino acid biosynthesis, carbohydrate metabolism, and central carbon metabolism and fermentation were more abundant in the intervention group. Other enhanced pathways were related to nucleotide biosynthesis, transcription, cell wall biosynthesis, and housekeeping metabolism (Figure 3). No differences in metabolic pathway abundances were observed in other time points.

**Figure 3.**
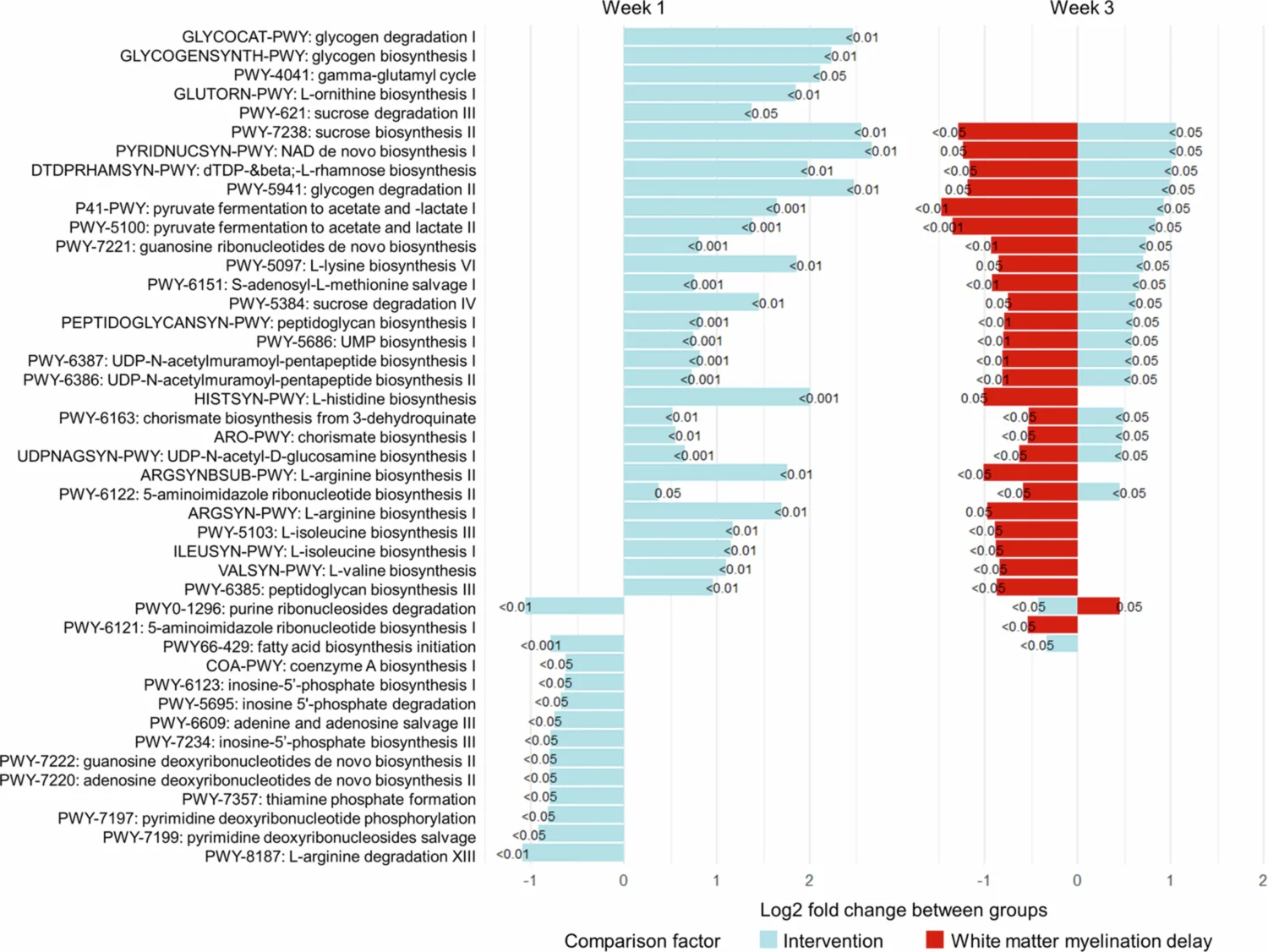
Differential abundance of metabolic pathways across conditions and time points. Bars represent the fold change in pathway abundance, and the colors indicate the type of comparison. Only pathways with significant differences are shown, with adjusted *P*-values shown as labels.

A comparison of the gut microbiome between infants with normal and delayed white matter myelination at TEA showed that white matter myelination delay was accompanied by reduced capacity for pyruvate fermentation in 3-week-old infants. Pathway II for pyruvate fermentation to acetate and lactate (PWY-5100) and pathway I for pyruvate fermentation to acetate and (S)-lactate (P41-PWY) (log2 change: 1.34; *P* = 4.93 × 10^−4^ and log2 change: 1.47; *P* = 0.003, respectively) showed the highest difference between the groups (Figure 3). Both pathways were enhanced in the intervention group: PWY-5100 at week 1 and 3, and P41-PWY at week 1 (Figure 3).

## Discussion

The gut microbiome of the preterm infants in the present study progressed through distinct community types during the early postnatal period, with *Bifidobacterium*-dominated communities representing a more mature stage that followed initial colonization by *Staphylococcus-, Enterococcus-,* and *Enterobacteriaceae-*rich communities, in agreement with studies following the preterm gut microbiome development.^18-21^ ^,^ ^23^ Supplementation with a mixture containing *B. breve* M-16V, scGOS/lcFOS, and L-glutamine facilitated the early establishment of the administered probiotic strain and accelerated gut microbiome maturation, similarly to studies investigating targeted probiotic use in preterm infants.^59-62^ HMOs were used to promote the colonization and growth of the probiotic strain^43^ and glutamine to support intestinal barrier integrity.^41^ ^,^ ^42^ Colonization was robust but transient, with significantly higher abundance in the intervention group during the supplementation period until 6 weeks after birth, despite the cross-colonization of the administered strain in a proportion of control infants. The supplementation resulted in higher *Bifidobacterium* levels accompanied by lower *Staphylococcus* and *Enterococcus* levels in the first 3 weeks of life.

Unlike what is commonly accepted for full-term infants,^63^ sex and delivery mode did not significantly influence the gut microbiome composition. Preterm infants have been associated with sparse, low-diversity initial gut microbiomes regardless of delivery mode, possibly reflecting clinical practices and physiological intestinal immaturity.^18^ ^,^ ^20^ Nutrition type also showed no effect on the microbiome structure, with infants fed with mother milk not showing significantly different microbiome structure compared to the infants fed predominantly with preterm formula. Although weeks of gestation are often linked to differences concerning the preterm gut microbiome,^19^ we also found no influence of gestational age, possibly because our cohort included only very and extremely preterm infants. In contrast to studies with a wider range of gestational ages, which often capture differences between moderate, very, and extremely preterm infants,^17^ ^,^ ^19^ our cohort included infants with a gestational age of 24–30 weeks, who were hospitalized in the same NICU environment. Similar to other trials with probiotics, intervention was the primary factor influencing the preterm microbiome.^60^ ^,^ ^61^

Furthermore, antibiotic administration had a significant effect on the microbiome composition, favoring *Enterobacter* only at week 3 and only within the control group. Studies have shown that antibiotic treatment likely promotes the growth of opportunistic microbes already resident in the infant gut and that these same microbes can then go on to have adverse health effects.^64^ Such opportunists can also be the early colonizers we detected in our study, such as *Enterobacteriaceae*, enterococci, and staphylococci.^65^ The low proportion of infants receiving antibiotics in the present study does not allow us to draw robust conclusions on the effect of antibiotics on the preterm gut microbiome development; however, the fact that antibiotics had an effect only in the control group warrants further investigation to test whether probiotics can prevent opportunistic pathogens from exploiting available niches in the infant gut after antibiotic administration.

Two phenotypes of brain injury diagnosed at TEA, white matter thinning of the corpus callosum and white matter myelination delay, were associated with early-life microbiome differences. White matter injury, which is the most common brain injury pattern in extremely preterm infants,^66^ is indicative of disrupted maturation and regional brain tissue volume reduction.^67^ Infants whose gut microbial communities were dominated by *Staphylococcus*, *Enterobacter*, and *Enterococcus* exhibited a higher occurrence of those brain injury phenotypes. This aligns with a prior study showing that 3-week-old preterm infants with *Enterococcus*- and *Staphylococcus*-dominated gut microbiome communities achieved lower developmental scores later in life.^24^ At the same time, we found that infants of the intervention group, as well as infants without signs of such encephalopathy, were characterized by higher *B. breve* abundances. This observation is consistent with accumulating evidence linking low early-life *Bifidobacterium* levels to adverse neurological outcomes in preterm infants.^18^ ^,^ ^68^ Taken together, these findings reinforce the concept that the first weeks after preterm birth are a critical window during which microbial colonization and key neurodevelopmental processes – such as white matter maturation, occur in parallel.^8^ Nutrition that can stimulate early colonization by beneficial microbes such as *B. breve* may therefore modulate gut–brain signaling, potentially protecting neurodevelopmental trajectories in the vulnerable preterm population.

The intestinal microbiome is increasingly recognized as a modifiable factor influencing the risk of encephalopathy in preterm newborns through the production of metabolites that participate in gut–brain axis signaling.^15^ ^,^ ^34^ In the present study, the metabolic shifts accompanying the intervention point to enhanced functional capacity in pathways linked to amino acid production, core carbon metabolism, and carbohydrate metabolism – processes that may have important implications for preterm infants by supporting nutrient provision, microbial energy metabolism, and metabolite-mediated gut–brain signaling. On the other hand, other affected pathways primarily related to microbial replication may reflect a microbiome state associated with favorable developmental conditions, rather than a direct effect on the host.

Notably, the metabolic pathways that differed the most between infants with and without white matter myelination delay at TEA were involved in pyruvate fermentation. These energy-producing metabolic pathways involved in the production of acetate and lactate were enriched in both the intervention group and in infants who did not exhibit white matter myelination delay. Pyruvate fermentation plays a central role in anaerobic energy metabolism by regenerating NAD⁺, supporting sustained glycolysis in low-oxygen environments. This is particularly relevant for preterm infants, whose tissues often face metabolic stress due to immature vascularization and a fluctuating oxygen supply. Metabolites derived from pyruvate fermentation, such as acetate, propanoate, and lactate, have been shown to downregulate pro-inflammatory responses in intestinal epithelial and myeloid cells,^69^ suggesting a potential mechanism whereby gut microbial metabolism could mitigate systemic inflammation. Acetate and propanoate have also been attributed the potential to affect brain structure and function,^70^ through (neuro)immune modulation.^71^ Oral administration of acetate in germ-free mice accelerated the maturation of microglia,^72^ while lactate has been shown to mediate brain neurogenesis and neuroprotection in mice.^73^ ^,^ ^74^ Thus, the enrichment of pyruvate fermentation pathways in both the intervention group and infants without brain injury highlights potential microbiome-mediated mechanisms of neuroprotection and warrants further investigation.^75^

The randomized controlled design of this study provided a clearly defined intervention window and minimized confounding related to the nutritional supplementation, facilitating the evaluation of its associations with preterm gut microbiome composition and functional capacity. Also, the intervention utilized a clinically relevant probiotic strain, previously shown to be safe for neonatal populations. Repeated sampling enabled detailed microbiome profiling, and integration of microbiome data with brain development markers provided a multidimensional view of potential gut–brain interactions. Finally, the incorporation of functional metagenomics offered insights into how the microbiome may influence brain development.

However, certain limitations should be noted. This study represents a secondary analysis of a randomized controlled trial;^38^ consequently, the original sample size calculation was based on the primary endpoint of the parent trial. Attrition between enrolment and data collection lowered the sample size of the initial cohort. In addition, this was a single-centre study. Consequently, our findings may partially reflect local clinical practices and patient characteristics, which may limit the generalizability of the results to other neonatal intensive care units. Also, the cross-colonization of the probiotic strain in the control group, which is often reported in probiotic trials conducted in neonatal units,^34^ ^,^ ^59^ ^,^ ^76^ may have masked the extent of the intervention effect. Furthermore, testing of additional probiotic strains within the *Bifidobacterium* genus, especially of strains that have exhibited beneficial impact on infant health,^60^ ^,^ ^77-79^ will provide more insight into whether the observed effects are strain-specific or represent a broader genus-level phenomenon. Finally, future studies integrating additional host and microbial functional data, such as metabolomic profiling of microbial metabolites and host immune markers, may provide greater mechanistic insight into the causal pathways linking the gut microbiome and neurodevelopment.

In conclusion, supplementation with a nutritional product containing *B. breve* M-16V, scGOS/lcFOS, and L-glutamine accelerated the establishment of gut microbial communities that could be associated with improved health outcomes. These gut communities showed elevated metabolic capacity centered around amino-acid synthesis, carbohydrate metabolism, and core carbon metabolism. Improved white matter development in extremely and very premature infants was associated with pyruvate fermentation pathways that were enhanced in the gut microbiome of the intervention group.

## Supplementary Material

Supplement2.xlsx

Supplement1.docx Supplement1.docx

## Data Availability

Complete taxonomic and functional microbiome compositional data on which statistical analysis was based are provided in Supplement 2. The underlying dataset is available under accession number EGAD50000002667 at https://ega-archive.org/datasets/EGAD50000002667. It contains personal data, and there is no legal approval to use the data for other purposes than those agreed upon by the participants.

## References

[1] Vogel JP , Chawanpaiboon S , Moller AB , Watananirun K , Bonet M , Lumbiganon P . The global epidemiology of preterm birth. Best Pract Res Clin Obstet Gynaecol. 2018;52:3–12. doi: 10.1016/j.bpobgyn.2018.04.003. 29779863

[2] Bhutta AT , Cleves MA , Casey PH , Cradock MM , Anand KJS . Cognitive and behavioral outcomes of school-aged children who were born preterm: a meta-analysis. J Am Med Assoc. 2002;288(6):728–737. doi: 10.1001/jama.288.6.728. 12169077

[3] Marlow N , Wolke D , Bracewell MA , Samara M . Neurologic and developmental disability at six years of age after extremely preterm birth. N Engl J Med. 2005;352(1):9–19. doi: 10.1056/NEJMoa041367. 15635108

[4] Dubois J , Benders M , Cachia A , Lazeyras F , Ha-Vinh Leuchter R , Sizonenko SV , Borradori-Tolsa C , Mangin JF , Huppi PS . Mapping the early cortical folding process in the preterm newborn brain. Cereb Cortex. 2008;18(6):1444–1454. doi: 10.1093/cercor/bhm180. 17934189

[5] Miller SP , Ferriero DM , Leonard C , Piecuch R , Glidden DV , Partridge JC , Perez M , Mukherjee P , Vigneron DB , Barkovich AJ . Early brain injury in premature newborns detected with magnetic resonance imaging is associated with adverse early neurodevelopmental outcome. J Pediatr. 2005;147(5):609–616. doi: 10.1016/j.jpeds.2005.06.033. 16291350

[6] Peterson BS , Vohr B , Staib LH , Cannistraci CJ , Dolberg A , Schneider KC , Katz KH , Westerveld M , Sparrow S , Anderson AW , et al. Regional brain volume abnormalities and long-term cognitive outcome in preterm infants. J Am Med Assoc. 2000;284(15):1939–1947. doi: 10.1001/jama.284.15.1939. 11035890

[7] Inder TE , Volpe JJ , Anderson PJ . Defining the neurologic consequences of preterm birth. N Engl J Med. 2023;389(5):441–453. doi: 10.1056/NEJMra2303347. 37530825

[8] Borre YE , O'Keeffe GW , Clarke G , O’Keeffe GW , Stanton C , Dinan TG , Cryan JF . Microbiota and neurodevelopmental Windows: implications for brain disorders. Trends Mol Med. 2014;20(9):509–518. doi: 10.1016/j.molmed.2014.05.002. 24956966

[9] Damiani F , Cornuti S , Tognini P . The gut-brain connection: exploring the influence of the gut microbiota on neuroplasticity and neurodevelopmental disorders. Neuropharmacology. 2023;231:109491. doi: 10.1016/j.neuropharm.2023.109491. 36924923

[10] Lu J , Claud EC . Connection between gut microbiome and brain development in preterm infants. Dev Psychobiol. 2019;61(5):739–751. doi: 10.1002/dev.21806. PMC6728148 30460694

[11] Stewart CJ , Embleton ND , Marrs ECL , Smith DP , Fofanova T , Nelson A , Skeath T , Perry JD , Petrosino JF , Berrington JE , et al. Longitudinal development of the gut microbiome and metabolome in preterm neonates with late onset sepsis and healthy controls. Microbiome. 2017;5(1):75. doi: 10.1186/s40168-017-0295-1. PMC5508794 28701177

[12] Stewart CJ , Embleton ND , Marrs ECL , Smith DP , Nelson A , Abdulkadir B , Skeath T , Petrosino JF , Perry JD , Berrington JE , et al. Temporal bacterial and metabolic development of the preterm gut reveals specific signatures in health and disease. Microbiome. 2016;4(1):67. doi: 10.1186/s40168-016-0216-8. PMC5200962 28034304

[13] Olm MR , Bhattacharya N , Crits-Christoph A , Firek BA , Baker R , Song YS , Morowitz MJ , Banfield JF . Necrotizing enterocolitis is preceded by increased gut bacterial replication, klebsiella, and fimbriae-encoding bacteria. Science Adv. 2019;5(12):5727–5738. doi: 10.1126/sciadv.aax5727. PMC6905865 31844663

[14] Pammi M , Cope J , Tarr PI , Warner BB , Morrow AL , Mai V , Gregory KE , Kroll JS , McMurtry V , Ferris MJ , et al. Intestinal dysbiosis in preterm infants preceding necrotizing enterocolitis: a systematic review and meta-analysis. Microbiome. 2017;5(1):31. doi: 10.1186/s40168-017-0248-8. PMC5343300 28274256

[15] Xiao J . Role of the gut microbiota-brain axis in brain damage in preterm infants. ACS Pharmacol Transl Sci. 2024;7(5):1197–1204. doi: 10.1021/acsptsci.3c00369. PMC11091980 38751622

[16] Bogaert D , van Beveren GJ , de Koff EM , Lusarreta Parga P , Balcazar Lopez CE , Koppensteiner L , Clerc M , Hasrat R , Arp K , Chu MLJ , et al. Mother-to-infant microbiota transmission and infant microbiota development across multiple body sites. Cell Host Microbe. 2023;31(3):447–460. doi: 10.1016/J.CHOM.2023.01.018. 36893737

[17] Chernikova DA , Madan JC , Housman ML , Zain-ul-abideen M , Lundgren SN , Morrison HG , Sogin ML , Williams SM , Moore JH , Karagas MR , et al. The premature infant gut microbiome during the first 6 weeks of life differs based on gestational maturity at birth. Pediatr Res. 2018;84(1):71–79. doi: 10.1038/s41390-018-0022-z. PMC6082716 29795209

[18] Dahl C , Stigum H , Valeur J , Iszatt N , Lenters V , Peddada S , Bjørnholt JV , Midtvedt T , Mandal S , Eggesbø M . Preterm infants have distinct microbiomes not explained by mode of delivery, breastfeeding duration or antibiotic exposure. Int J Epidemiol. 2018;47(5):1658–1669. doi: 10.1093/ije/dyy064. 29688458

[19] Korpela K , Blakstad EW , Moltu SJ , Strømmen K , Nakstad B , Rønnestad AE , Brække K , Iversen PO , Drevon CA , de Vos W . Intestinal microbiota development and gestational age in preterm neonates. Sci Rep. 2018;8(1):1–9. doi: 10.1038/s41598-018-20827-x. PMC5802739 29410448

[20] Stewart CJ , Embleton ND , Clements E , Luna PN , Smith DP , Fofanova TY , Nelson A , Taylor G , Orr CH , Petrosino JF , et al. Cesarean or vaginal birth does not impact the longitudinal development of the gut microbiome in a cohort of exclusively preterm infants. Front Microbiol. 2017;8:1008. doi: 10.3389/fmicb.2017.01008. PMC5459931 28634475

[21] La Rosa PS , Warner BB , Zhou Y , Weinstock GM , Sodergren E , Hall-Moore CM , Stevens HJ , Bennett WE , Shaikh N , Linneman LA , et al. Patterned progression of bacterial populations in the premature infant gut. Proc Natl Acad Sci USA. 2014;111(34):12522–12527. doi: 10.1073/pnas.1409497111. PMC4151715 25114261

[22] Beghetti I , Barone M , Turroni S , Biagi E , Sansavini A , Brigidi P , Corvaglia L , Aceti A . Early-life gut microbiota and neurodevelopment in preterm infants: any role for bifidobacterium? Eur J Pediatr. 2021;181:1773–1777. doi: 10.1007/s00431-021-04327-1. 34842970

[23] Liu X , Cong X , Li G , Maas K , Chen K . Multivariate log-contrast regression with sub-compositional predictors: testing the association between preterm infants’ gut microbiome and neurobehavioral outcomes. Stat Med. 2022;41:580–594. doi: 10.1002/SIM.9273. 34897772

[24] Rozé JC , Ancel PY , Marchand-Martin L , Rousseau C , Montassier E , Monot C , Le Roux K , Butin M , Resche-Rigon M , Aires J , et al. Assessment of neonatal intensive care unit practices and preterm newborn gut microbiota and 2-Year neurodevelopmental outcomes. JAMA Netw Open. 2020;3(9):e2018119. doi: 10.1001/jamanetworkopen.2020.18119. PMC7512059 32965499

[25] Seki D , Mayer M , Hausmann B , Pjevac P , Giordano V , Goeral K , Unterasinger L , Klebermaß-Schrehof K , De Paepe K , Van de Wiele T , et al. Aberrant gut-microbiota-immune-brain axis development in premature neonates with brain damage. Cell Host Microbe. 2021;29:1–15. doi: 10.1016/j.chom.2021.08.004. PMC8525911 34480872

[26] Ma J , Li Z , Zhang W , Mei H , Zhuo N , Wang H , Wu D . Comparison of gut microbiota in exclusively breast-fed and formula-fed babies: a study of 91 term infants. Sci Rep. 2020;10(1):1–11. doi: 10.1038/s41598-020-72635-x. PMC7519658 32978424

[27] Masi AC , Stewart CJ . Untangling human milk oligosaccharides and infant gut microbiome. iSci. 2022;25(1):103542. doi: 10.1016/J.ISCI.2021.103542. PMC8671521 34950861

[28] Stewart CJ , Ajami NJ , O'Brien JL , O’Brien JL , Hutchinson DS , Smith DP , Wong MC , Ross MC , Lloyd RE , Doddapaneni H , et al. Temporal development of the gut microbiome in early childhood from the TEDDY study. Natur. 2018;562(7728):583–588. doi: 10.1038/s41586-018-0617-x. PMC6415775 30356187

[29] Henderickx JGE , Zwittink RD , van Lingen RA , Knol J , Belzer C . The preterm gut microbiota: an inconspicuous challenge in nutritional neonatal care. Front Cell Infect Microbiol. 2019;9:85. doi: 10.3389/fcimb.2019.00085. PMC6454191 31001489

[30] Henderickx JGE , Zwittink RD , Renes IB , van Lingen RA , van Zoeren-Grobben D , Jebbink LJG , Boeren S , van Elburg RM , Knol J , Belzer C . Maturation of the preterm gastrointestinal tract can be defined by host and microbial markers for digestion and barrier defense. Sci Rep. 2021;11(1):12808. doi: 10.1038/s41598-021-92222-y. PMC8211855 34140588

[31] Gasparrini AJ , Wang B , Sun X , Kennedy EA , Hernandez-Leyva A , Ndao IM , Tarr PI , Warner BB , Dantas G . Persistent metagenomics signatures of early-life hospitalization and antibiotic treatment in the infant gut microbiota and resistome. Nat Microbiol. 2019;4(12):2285–2297. doi: 10.1038/s41564-019-0550-2. PMC6879825 31501537

[32] Zwittink RD , van Zoeren-Grobben D , Martin R , van Lingen RA , Groot Jebbink LJ , Boeren S , Renes IB , van Elburg RM , Belzer C , Knol J . Metaproteomics reveals functional differences in intestinal microbiota development of preterm infants. Mol Cell Proteomics. 2017;16(9):1610–1620. doi: 10.1074/mcp.RA117.000102. PMC5587861 28684633

[33] Buffet-Bataillon S , Bellanger A , Boudry G , Gangneux J , Yverneau M , Beuchée A , Blat S , Le Huërou-Luron I . New insights into microbiota modulation-based nutritional interventions for neurodevelopmental outcomes in preterm infants. Front Microbiol. 2021;12:676622. doi: 10.3389/fmicb.2021.676622. PMC8232935 34177860

[34] Vaher K , Cabez MB , Parga PL , Binkowska J , van Beveren GJ , Odendaal M , Sullivan G , Stoye DQ , Corrigan A , Quigley AJ , et al. The neonatal gut microbiota: a role in the encephalopathy of prematurity. Cell Reports Medicine. 2024;5(12):101845. doi: 10.1016/j.xcrm.2024.101845. PMC11722115 39637857

[35] Costeloe K , Hardy P , Juszczak E , Wilks M , Millar MR . Bifidobacterium breve BBG-001 in very preterm infants: a randomised controlled phase 3 trial. Lancet. 2016;387:649–660. doi: 10.1016/S0140-6736(15)01027-2. 26628328

[36] Robertson C , Robertson C , Savva GM , Clapuci R , Jones J , Maimouni H , Brown E , Minocha A , Hall LJ , Clarke P . Incidence of necrotising enterocolitis before and after introducing routine prophylactic lactobacillus and bifidobacterium probiotics. Arch Dis Child Fetal Neonatal Ed. 2020;105(4):380–386. doi: 10.1136/ARCHDISCHILD-2019-317346. PMC7363787 31666311

[37] van den Akker CHP , van Goudoever JB , Szajewska H , Embleton ND , Hojsak I , Reid D , Shamir R . ESPGHAN working group for probiotics, prebiotics & committee on nutrition. Probiotics for preterm infants: a strain-specific systematic review and network meta-analysis. J Pediatr Gastroenterol Nutr. 2018;67(1):103–122. doi: 10.1097/MPG.0000000000001897. 29384838

[38] Hortensius LM , van den Hooven EH , Dudink J , Tataranno ML , van Elburg RM , Benders MJNL . NutriBrain: protocol for a randomised, double-blind, controlled trial to evaluate the effects of a nutritional product on brain integrity in preterm infants. BMC Pediatr. 2021;21(1):132. doi: 10.1186/s12887-021-02570-x. PMC7968155 33731062

[39] Patole S , Keil AD , Chang A , Nathan E , Doherty D , Simmer K , Esvaran M , Conway P , Cameron DW . Effect of bifidobacterium breve M-16V supplementation on fecal bifidobacteria in preterm neonates--a randomised double blind placebo controlled trial. PLoS One. 2014;9(3):e89511. doi: 10.1371/journal.pone.0089511. PMC3940439 24594833

[40] Wang C , Shoji H , Sato H , Nagata S , Ohtsuka Y , Shimizu T , Yamashiro Y . Effects of oral administration of bifidobacterium breve on fecal lactic acid and short-chain fatty acids in low birth weight infants. J Pediatr Gastroenterol Nutr. 2007;44(2):252–257. doi: 10.1097/01.mpg.0000252184.89922.5f. 17255840

[41] van den Berg A , van Elburg RM , Westerbeek EA , Twisk JW , Fetter WP . Glutamine-enriched enteral nutrition in very-low-birth-weight infants and effects on feeding tolerance and infectious morbidity: a randomized controlled trial. AJCN. 2005;81(6):1397–1404. doi: 10.1093/ajcn/81.6.1397. 15941893

[42] Vaughn P , Thomas P , Clark R , Neu J . Enteral glutamine supplementation and morbidity in low birth weight infants. J Pediatr. 2003;142(6):662–668. doi: 10.1067/mpd.2003.208. 12838195

[43] Modi N , Uthaya S , Fell J , Kulinskaya E . A randomized, double-blind, controlled trial of the effect of prebiotic oligosaccharides on enteral tolerance in preterm infants (ISRCTN77444690). Pediatr Res. 2010;68(5):440–445. doi: 10.1203/PDR.0b013e3181f1cd59. 20639792

[44] van Beek PE , Claessens NHP , Makropoulos A , Groenendaal F , de Vries LS , Counsell SJ , Benders MJ . Increase in brain volumes after implementation of a nutrition regimen in infants born extremely preterm. J Pediatr. 2020;223:57–63.e5. doi: 10.1016/j.jpeds.2020.04.063. 32389719

[45] Kidokoro H , Neil JJ , Inder TE . New MR imaging assessment tool to define brain abnormalities in very preterm infants at term. Am J Neuroradiol. 2013;34(11):2208–2214. doi: 10.3174/ajnr.A3521. PMC4163698 23620070

[46] Bolger AM , Lohse M , Usadel B . Trimmomatic: a flexible trimmer for illumina sequence data. Bioinformatics. 2014;30(15):2114–2120. doi: 10.1093/bioinformatics/btu170. PMC4103590 24695404

[47] Langmead B , Salzberg SL . Fast gapped-read alignment with bowtie 2. Nat Methods. 2012;9(4):357–359. doi: 10.1038/nmeth.1923. PMC3322381 22388286

[48] Truong DT , Tett A , Pasolli E , Huttenhower C , Segata N . Microbial strain-level population structure and genetic diversity from metagenomes. Genome Res. 2017;27(4):626–638. doi: 10.1101/gr.216242.116. PMC5378180 28167665

[49] Segata N , Waldron L , Ballarini A , Narasimhan V , Jousson O , Huttenhower C . Metagenomic microbial community profiling using unique clade-specific marker genes. Nat Methods. 2012;9(8):811–814. doi: 10.1038/nmeth.2066. PMC3443552 22688413

[50] Beghini F , McIver LJ , Blanco-Míguez A , Dubois L , Asnicar F , Maharjan S , Mailyan A , Manghi P , Scholz M , Thomas AM , et al. Integrating taxonomic, functional, and strain-level profiling of diverse microbial communities with bioBakery 3. eLife. 2021;10:e65088. doi: 10.7554/eLife.65088. PMC8096432 33944776

[51] R Core Team. R: A language and environment for statistical computing. R Foundation for Statistical Computing. 2021. https://www.R-project.org.

[52] Oksanen J , Simpson G , Blanchet F , Kindt R , Legendre P , Minchin P , O'Hara R , Solymos P , Stevens M , Szoecs E . Vegan: Community Ecology Package. 2026. doi: 10.32614/CRAN.package.vegan.

[53] Rivera-Pinto J , Egozcue JJ , Pawlowsky-Glahn V , Paredes R , Noguera-Julian M , Calle ML , Lozupone C . Balances: a new perspective for microbiome analysis. mSystems. 2018;3(4):e00053–18. doi: 10.1128/msystems.00053-18. PMC6050633 30035234

[54] De Cáceres M , Legendre P . Associations between species and groups of sites: indices and statistical inference. Ecology. 2009;90(12):3566–3574. doi: 10.1890/08-1823.1. 20120823

[55] Love MI , Huber W , Anders S . Moderated estimation of fold change and dispersion for RNA-seq data with DESeq2. Genome Biol. 2014;15:550. doi: 10.1186/s13059-014-0550-8. PMC4302049 25516281

[56] Murtagh F , Legendre P . Ward’s hierarchical agglomerative clustering method: which algorithms implement ward’s criterion? J Classif. 2014;31(3):274–295. doi: 10.1007/s00357-014-9161-z.

[57] Suzuki R , Shimodaira H . Pvclust: an R package for assessing the uncertainty in hierarchical clustering. Bioinformatics. 2006;22(12):1540–1542. doi: 10.1093/bioinformatics/btl117. 16595560

[58] Zeileis A , Meyer D , Hornik K . Residual-based shadings for visualizing (conditional) independence. J Comput Graph Stat. 2007;16(3):507–525. doi: 10.1198/106186007X237856.

[59] Samara J , Moossavi S , Alshaikh B , Ortega VA , Pettersen VK , Ferdous T , Hoops SL , Soraisham A , Vayalumkal J , Dersch-Mills D , et al. Supplementation with a probiotic mixture accelerates gut microbiome maturation and reduces intestinal inflammation in extremely preterm infants. Cell Host Microbe. 2022;30(5):696–711.e5. doi: 10.1016/j.chom.2022.04.005. 35550672

[60] Beck LC , Masi AC , Young GR , Vatanen T , Lamb CA , Smith R , Coxhead J , Butler A , Marsland BJ , Embleton ND , et al. Strain-specific impacts of probiotics are a significant driver of gut microbiome development in very preterm infants. Nat Microbiol. 2022;7:1525–1535. doi: 10.1038/s41564-022-01213-w. PMC9519454 36163498

[61] He P , Yu L , Tian F , Chen W , Zhang H , Zhai Q . Effects of probiotics on preterm infant gut microbiota across populations: a systematic review and meta-analysis. Adv Nutr. 2024;15(6):100233. doi: 10.1016/j.advnut.2024.100233. PMC11251410 38908894

[62] Rao C , Coyte KZ , Bainter W , Geha RS , Martin CR , Rakoff-Nahoum S . Multi-kingdom ecological drivers of microbiota assembly in preterm infants. Natur. 2021;591:633–638. doi: 10.1038/s41586-021-03241-8. PMC7990694 33627867

[63] Bokulich NA , Chung J , Battaglia T , Henderson N , Jay M , Li H , Lieber A , Wu F , Perez-Perez GI , Chen Y , et al. Antibiotics, birth mode, and diet shape microbiome maturation during early life. Sci Transl Med. 2016;8(343):343–382. doi: 10.1126/scitranslmed.aad7121. PMC5308924 27306664

[64] Schwartz DJ , Shalon N , Wardenburg K , DeVeaux A , Wallace MA , Hall-Moore C , Ndao IM , Sullivan JE , Radmacher P , Escobedo M , et al. Gut pathogen colonization precedes bloodstream infection in the neonatal intensive care unit. Sci Transl Med. 2023;15(694):eadg5562. doi: 10.1126/scitranslmed.adg5562. PMC10259202 37134153

[65] Thänert R , Schwartz DJ , Keen EC , Hall-Moore C , Wang B , Shaikh N , Ning J , Rouggly-Nickless LC , Thänert A , Ferreiro A , et al. Clinical sequelae of gut microbiome development and disruption in hospitalized preterm infants. Cell Host Microbe. 2024;32(10):1822–1837.e5. doi: 10.1016/j.chom.2024.07.027. Erratum in: Cell Host Microbe;32(10):1868-1869. doi: 39197454. PMC11466706

[66] Inder TE , Wells SJ , Mogridge NB , Spencer C , Volpe JJ . Defining the nature of the cerebral abnormalities in the premature infant: a qualitative magnetic resonance imaging study. J Pediatr. 2003;143(2):171–179. doi: 10.1067/S0022-3476(03)00357-3. 12970628

[67] Counsell SJ , Allsop JM , Harrison MC , Larkman DJ , Kennea NL , Kapellou O , Cowan FM , Hajnal JV , Edwards AD , Rutherford MA . Diffusion-weighted imaging of the brain in preterm infants with focal and diffuse White matter abnormality. Pediatrics. 2003;112(1):1–7. doi: 10.1542/peds.112.1.1. 12837859

[68] Van den Berg JP , Westerbeek EA , Bröring-Starre T , Bröring‐Starre T , Garssen J , van Elburg RM . Neurodevelopment of preterm infants at 24 months after neonatal supplementation of a prebiotic mix: a randomized trial. J Pediatr Gastroenterol Nutr. 2016;63(2):270–276. doi: 10.1097/MPG.0000000000001148. 26859091

[69] Iraporda C , Errea A , Romanin DE , Cayet D , Pereyra E , Pignataro O , Sirard JC , Garrote GL , Abraham AG , Rumbo M . Lactate and short chain fatty acids produced by microbial fermentation downregulate proinflammatory responses in intestinal epithelial cells and myeloid cells. Immunobiology. 2015;220(10):1161–1169. doi: 10.1016/j.imbio.2015.06.004. 26101138

[70] O'Riordan KJ , Collins MK , Moloney GM , Knox EG , Aburto MR , Fülling C , Morley SJ , Clarke G , Schellekens H , Cryan JF . Short chain fatty acids: microbial metabolites for gut-brain axis signaling. Mol Cell Endocrinol. 2022;546:111572. doi: 10.1016/j.mce.2022.111572. 35066114

[71] Ratajczak W , Rył A , Mizerski A , Walczakiewicz K , Sipak O , Laszczyńska M . Immunomodulatory potential of gut microbiome-derived short-chain fatty acids (SCFAs). Acta Biochim Pol. 2019;66(1):1–12. doi: 10.18388/abp.2018_2648. 30831575

[72] Erny D , Hrabě de Angelis AL , Jaitin D , Wieghofer P , Staszewski O , David E , Keren-Shaul H , Mahlakoiv T , Jakobshagen K , Buch T , et al. Host microbiota constantly control maturation and function of microglia in the CNS. Nat Neurosci. 2015;18(7):965–977. doi: 10.1038/nn.4030. PMC5528863 26030851

[73] Berthet C , Castillo X , Magistretti PJ , Hirt L . New evidence of neuroprotection by lactate after transient focal cerebral ischemia: extended benefit after intracerebroventricular injection and efficacy of intravenous administration. Cerebrovasc Dis. 2012;34(5):329–335. doi: 10.1159/000343657. 23154656

[74] Lambertus M , Øverberg LT , Andersson KA , Hjelden MS , Hadzic A , Haugen ØP , Storm‐Mathisen J , Bergersen LH , Geiseler S , Morland C . L-lactate induces neurogenesis in the mouse ventricular-subventricular zone via the lactate receptor HCA1. Acta Physiol (Oxf). 2021;231(3):e13587. doi: 10.1111/apha.13587. 33244894

[75] He Y , Zhang Y , Li F , Shi Y . White matter injury in preterm infants: pathogenesis and potential therapy from the aspect of the gut-brain axis. Front Neurosci. 2022;16:849372. doi: 10.3389/fnins.2022.849372. PMC9099073 35573292

[76] Hickey L , Garland SM , Jacobs SE , O'Donnell CPF , Tabrizi SN . Cross-colonization of infants with probiotic organisms in a neonatal unit. J Hosp Infect. 2014;88(3):226–229. doi: 10.1016/j.jhin.2014.09.006. 25447201

[77] Henrick BM , Rodriguez L , Lakshmikanth T , Pou C , Henckel E , Arzoomand A , Olin A , Wang J , Mikes J , Tan Z , et al. Bifidobacteria-mediated immune system imprinting early in life. Cell. 2021;184(15):3884–3898.e11. doi: 10.1016/j.cell.2021.05.030. 34143954

[78] Xu C , Odamaki T , Hiraku A , Nakata S , Arai S , Iwabuchi N , Tanaka M , Tsuno T , Nakamura M . Anti-inflammatory effects of *bifidobacterium infantis* M-63 during the early postnatal period in term infants. Pediatr Res. 2026;99(5):1842–1850. doi: 10.1038/s41390-025-04263-y. PMC13221305 40681696

[79] Yang S , Cai J , Su Q , Li Q , Meng X . Human milk oligosaccharides combine with *bifidobacterium longum* to form the “golden shield” of the infant intestine: metabolic strategies, health effects, and mechanisms of action. Gut Microbes. 2024;16(1):2430418. doi: 10.1080/19490976.2024.2430418. PMC11587862 39572856

