# Supplementary material for: Synbiotic supplementation leads to gut microbiome communities with functional capacity linked to better brain white matter development in preterm infants
Source: Gut Microbes. 2026 Oct 7;18(1):2743949. doi: 10.1080/19490976.2026.2743949 (PMC13656805; doi:10.1080/19490976.2026.2743949)
Supplement: Supplementary file 1 [file KGMI_A_2743949_SM9945.docx]

Supplemental Content

**Synbiotic supplementation leads to gut microbiome communities with functional capacity linked to better brain white matter development in preterm infants**

**Figure S1.** Flow of participants through enrollment, randomization, intervention allocation, follow-up, and inclusion in analyses. A total of 99 preterm infants were randomized to the intervention or control group. Microbiome data were obtained from stool samples collected at multiple time points. For a subset of participants, integration of microbiome and brain development data was performed *post hoc*.

**Table S1.** Subjects with sequenced metagenomes per sampling point and available Kidokoro scores. Corrected age is calculated by subtracting the number of weeks the infant was born early from their chronological age to account for prematurity

| Planned sampling points | Sampling points in analysis | Subjects with sequenced metagenome | Subjects with Kidokoro scores assessed with MRI |
| --- | --- | --- | --- |
| Day 3 | Day 3 | 23 | 19 |
| Week 1 | Week 1 | 70 | 63 |
| Week 3 | Week 3 | 73 | 65 |
| Week 6 | Week 6 | 66 | 60 |
| Week 6- Week 36  depending on availability and hospital discharge | Weeks 7-8 | 32 | 29 |
|  | Weeks 8-12 | 43 | 39 |
|  | Weeks 12-18 | 48 | 45 |
|  | Weeks 18-36 | 42 | 41 |
| Term-equivalent age | Weeks 36- 42 | 12 | 10 |
| 4 months Corrected Age | 4 months Corrected Age | 6 | 6 |
| 15 months Corrected Age | 15 months Corrected Age | 32 | 30 |
| 24 months Corrected Age | 24 months Corrected Age | 26 | 22 |

**Table S2**. Baseline characteristics of study participants with sampled metagenomes at each sampling time point and per study group.

| Sampling points in analysis | Metadata | Subjects with sequenced metagenome | | Subjects with Kidokoro scores assessed with MRI | |
| --- | --- | --- | --- | --- | --- |
| Day 3 | Assignment to groups | Intervention: 14 | Control: 9 | Intervention: 12 | Control: 7 |
|  | Sex: male (%) | 42.86 | 66.67 | 41.67 | 71.43 |
|  | Vaginal delivery (%) | 50.00 | 33.33 | 50.00 | 14.29 |
|  | Nutrition with >80 mother milk (%) | 85.71 | 77.78 | 83.33 | 71.43 |
|  | Weeks of gestation average | 27.51 | 27.52 | 27.38 | 27.24 |
|  | Birth weight in grams average | 891.43 | 1069.44 | 891.67 | 1038.57 |
| Week 1 | Assignment to groups | Intervention: 37 | Control: 33 | Intervention: 32 | Control: 31 |
|  | Sex: male (%) | 64.86 | 63.64 | 67.74 | 64.52 |
|  | Vaginal delivery (%) | 45.95 | 39.39 | 46.88 | 35.48 |
|  | Nutrition with >80 mother milk (%) | 78.38 | 72.73 | 78.13 | 70.97 |
|  | Weeks of gestation average | 27.86 | 27.56 | 27.83 | 27.50 |
|  | Birth weight in grams average | 1027.51 | 1090.45 | 1033.81 | 1084.84 |
| Week 3 | Assignment to groups | Intervention: 37 | Control: 36 | Intervention: 32 | Control: 33 |
|  | Sex: male (%) | 56.76 | 63.89 | 56.25 | 63.64 |
|  | Vaginal delivery (%) | 40.54 | 44.44 | 40.63 | 39.39 |
|  | Nutrition with >80 mother milk (%) | 75.68 | 75.00 | 75.00 | 72.73 |
|  | Weeks of gestation average | 27.66 | 27.40 | 27.60 | 27.39 |
|  | Birth weight in grams average | 970.41 | 1065.83 | 967.78 | 1064.09 |
| Week 6 | Assignment to groups | Intervention: 33 | Control: 33 | Intervention: 29 | Control: 31 |
|  | Sex: male (%) | 66.67 | 60.61 | 65.52 | 58.06 |
|  | Vaginal delivery (%) | 36.36 | 42.42 | 37.93 | 38.71 |
|  | Nutrition with >80 mother milk (%) | 72.73 | 72.73 | 72.41 | 70.97 |
|  | Weeks of gestation average | 27.49 | 27.47 | 27.45 | 27.50 |
|  | Birth weight in grams average | 958.91 | 1074.24 | 960.62 | 1072.10 |
| Weeks 7-8 | Assignment to groups | Intervention: 15 | Control: 17 | Intervention: 14 | Control: 15 |
|  | Sex: male (%) | 60.00 | 58.82 | 64.29 | 66.67 |
|  | Vaginal delivery (%) | 40.00 | 35.29 | 42.86 | 26.67 |
|  | Nutrition with >80 mother milk (%) | 86.67 | 58.82 | 85.71 | 53.33 |
|  | Weeks of gestation average | 28.18 | 27.89 | 28.12 | 28.01 |
|  | Birth weight in grams average | 1104.20 | 1028.24 | 1096.29 | 1017.67 |
| Weeks 8-12 | Assignment to groups | Intervention: 22 | Control: 21 | Intervention: 20 | Control: 19 |
|  | Sex: male (%) | 72.73 | 61.90 | 70.00 | 57.89 |
|  | Vaginal delivery (%) | 45.45 | 42.86 | 45.00 | 36.84 |
|  | Nutrition with >80 mother milk (%) | 63.64 | 85.71 | 65.00 | 84.21 |
|  | Weeks of gestation average | 26.96 | 26.65 | 26.94 | 26.62 |
|  | Birth weight in grams average | 898.14 | 959.52 | 912.70 | 943.95 |
| Weeks 12-18 | Assignment to groups | Intervention: 25 | Control: 23 | Intervention: 23 | Control: 22 |
|  | Sex: male (%) | 68.00 | 65.22 | 65.22 | 68.18 |
|  | Vaginal delivery (%) | 48.00 | 65.22 | 47.83 | 36.36 |
|  | Nutrition with >80 mother milk (%) | 72.00 | 78.26 | 73.91 | 77.27 |
|  | Weeks of gestation average | 27.61 | 27.29 | 27.63 | 27.23 |
|  | Birth weight in grams average | 983.44 | 1011.96 | 984.57 | 1010.68 |
| Weeks 18-36 | Assignment to groups | Intervention: 21 | Control: 21 | Intervention: 21 | Control: 20 |
|  | Sex: male (%) | 71.43 | 61.90 | 71.43 | 65.00 |
|  | Vaginal delivery (%) | 52.38 | 23.81 | 52.38 | 20.00 |
|  | Nutrition with >80 mother milk (%) | 57.14 | 61.90 | 57.14 | 60.00 |
|  | Weeks of gestation average | 28.12 | 27.90 | 28.12 | 2786 |
|  | Birth weight in grams average | 1034.67 | 1090.00 | 1034.67 | 1092.50 |
| Weeks 36- 42 | Assignment to groups | Intervention: 8 | Control: 4 | Intervention: 7 | Control: 3 |
|  | Sex: male (%) | 75.00 | 75.00 | 71 | 67 |
|  | Vaginal delivery (%) | 62.50 | 75.00 | 71 | 67 |
|  | Nutrition with >80 mother milk (%) | 75.00 | 100.00 | 71 | 100 |
|  | Weeks of gestation average | 27.16 | 26.71 | 27.08 | 27.05 |
|  | Birth weight in grams average | 930.00 | 1092.50 | 972.86 | 1156.67 |
| 4 months Corrected Age | Assignment to groups | Intervention: 2 | Control: 4 | Intervention: 2 | Control: 4 |
|  | Sex: male (%) | 0.00 | 50.00 | 0.00 | 50.00 |
|  | Vaginal delivery (%) | 50.00 | 25.00 | 50.00 | 25.00 |
|  | Nutrition with >80 mother milk (%) | 100.00 | 50.00 | 100.00 | 50.00 |
|  | Weeks of gestation average | 27.57 | 27.07 | 27.57 | 27.07 |
|  | Birth weight in grams average | 940.00 | 1082.50 | 940.00 | 1082.50 |
| 15 months Corrected Age | Assignment to groups | Intervention: 16 | Control: 16 | Intervention: 15 | Control: 15 |
|  | Sex: male (%) | 62.50 | 68.75 | 66.67 | 66.67 |
|  | Vaginal delivery (%) | 56.25 | 25.00 | 53.33 | 20.00 |
|  | Nutrition with >80 mother milk (%) | 68.75 | 75.00 | 66.67 | 73.33 |
|  | Weeks of gestation average | 27.75 | 27.58 | 27.68 | 27.70 |
|  | Birth weight in grams average | 1014.94 | 1010.63 | 1005.93 | 1018.00 |
| 24 months Corrected Age | Assignment to groups | Intervention: 14 | Control: 12 | Intervention: 12 | Control: 10 |
|  | Sex: male (%) | 64.29 | 66.67 | 75.00 | 70.00 |
|  | Vaginal delivery (%) | 50.00 | 25.00 | 50.00 | 10.00 |
|  | Nutrition with >80 mother milk (%) | 57.14 | 75.00 | 50.00 | 70.00 |
|  | Weeks of gestation average | 27.71 | 27.89 | 27.51 | 28.03 |
|  | Birth weight in grams average | 1004.50 | 1009.17 | 974.83 | 1017.00 |

**Figure S2.** Relative abundance of total *Bifidobacterium breve* and *B. breve* M-16V across the study period until 36 weeks PMA. Boxplots show respective relative abundances (%) in the control and intervention groups at each sampling time point. Boxes represent the interquartile range, the horizontal line indicates the median, and whiskers extend to 1.5 × IQR. Wilcoxon rank sum tests compare abundances between groups.

**Figure S3.** Microbial composition of all fecal samples from preterm infants taken at 12 sampling time points, summarized at the taxonomic family level. Each bar within a sampling time point represent the microbial composition for both the intervention and the control group, respectively.

**Figure S4.** A) Gut microbial composition of all fecal samples taken at week 3 after birth for both the intervention and the control group, divided into infants receiving and not receiving antibiotics at the time of or one week prior to sampling. B) Relative abundance of the most abundant bacterial genera at week 3 after birth for both the intervention and the control group. Boxplots represent the overall genus abundance with the horizontal line at the median value. Wilcoxon rank sum tests compare abundances between infants receiving and not receiving antibiotics at the time of or one week prior to sampling.

**Figure S5.** Measurements of infants’ weight (g) from birth until the 60^th^ day after birth. No differences were observed between the intervention and control group after group comparison using linear mixed-effects models with group, feeding type, and sex as fixed effects, and infants as a random effect to account for repeated measurements.

**Table S3**. Indicative bacterial species for the gut microbiome communities of preterm infants belonging to the control or the intervention group and to preterm infants with or without white matter thinning of the corpus callosum and white matter myelination delay at one and three weeks after birth, respectively.

| Category | | Indicator species | indicVal | *P* |
| --- | --- | --- | --- | --- |
| Week 1 | | | | |
| Intervention | 0 | *Enterococcus faecalis* | 0.36 | 0.004 |
|  |  | *Staphylococcus epidermidis* | 0.28 | 0.001 |
|  |  | *Corynebacterium kefirresidentii* | 0.18 | 0.184 |
|  |  | *Staphylococcus hominis* | 0.18 | 0.482 |
|  |  | *Corynebacterium aurimucosum* | 0.14 | 0.419 |
|  | 1 | *Bifidobacterium breve* | 0.67 | 0.001 |
| White matter thinning of the corpus callosum | 0 | *Bifidobacterium breve* | 0.37 | 0.035 |
|  | 1 | *Staphylococcus haemolyticus* | 0.50 | 0.001 |
|  |  | *Staphylococcus epidermidis* | 0.32 | 0.007 |
|  |  | *Staphylococcus capitis* | 0.27 | 0.028 |
| Week 3 | | | | |
| Intervention | 0 | *Enterococcus faecalis* | 0.289 | 0.013 |
|  | 1 | *Bifidobacterium breve* | 0.30 | 0.015 |
|  |  | *Veillonella atypica* | 0.15 | 0.036 |
| White matter myelination delay | 0 | *Bifidobacterium breve* | 0.42 | 0.002 |
|  | 1 | *Varibaculum cambriense* | 0.26 | 0.026 |
|  |  | *Enterobacter roggenkampii* | 0.24 | 0.007 |
|  |  | *Acinetobacter johnsonii* | 0.22 | 0.047 |

**Figure S6.** A) Composition of preterm gut community types at genus level. B) Association plots of community type transition with the intervention, and with two white matter phenotypes: myelination delay and thinning of the corpus callosum. The circle size at week 3 is proportional to the number of subjects following the respective community transition. Color shows how much the observed frequency deviates from what would be expected under independence, with red indicating negative and blue indicating positive associations. Bar height is proportional to association significance and width is proportional to the support from data frequency.
